# Microbial Resistance to Antibiotics and Effective Antibiotherapy

**DOI:** 10.3390/biomedicines10051121

**Published:** 2022-05-12

**Authors:** Adriana Aurelia Chiș, Luca Liviu Rus, Claudiu Morgovan, Anca Maria Arseniu, Adina Frum, Andreea Loredana Vonica-Țincu, Felicia Gabriela Gligor, Maria Lucia Mureșan, Carmen Maximiliana Dobrea

**Affiliations:** Preclinical Department, Faculty of Medicine, “Lucian Blaga” University of Sibiu, 550169 Sibiu, Romania; adriana.chis@ulbsibiu.ro (A.A.C.); liviu.rus@ulbsibiu.ro (L.L.R.); claudiu.morgovan@ulbsibiu.ro (C.M.); loredana.vonica@ulbsibiu.ro (A.L.V.-Ț.); felicia.gligor@ulbsibiu.ro (F.G.G.); maria.muresan@ulbsibiu.ro (M.L.M.); carmen.dobrea@ulbsibiu.ro (C.M.D.)

**Keywords:** antibiotic resistance, multidrug-resistant bacteria, mechanism of resistance, persistence, biofilms, antibiotherapy

## Abstract

Currently, the efficacy of antibiotics is severely affected by the emergence of the antimicrobial resistance phenomenon, leading to increased morbidity and mortality worldwide. Multidrug-resistant pathogens are found not only in hospital settings, but also in the community, and are considered one of the biggest public health concerns. The main mechanisms by which bacteria develop resistance to antibiotics include changes in the drug target, prevention of entering the cell, elimination through efflux pumps or inactivation of drugs. A better understanding and prediction of resistance patterns of a pathogen will lead to a better selection of active antibiotics for the treatment of multidrug-resistant infections.

## 1. Introduction

**Figure 1:**
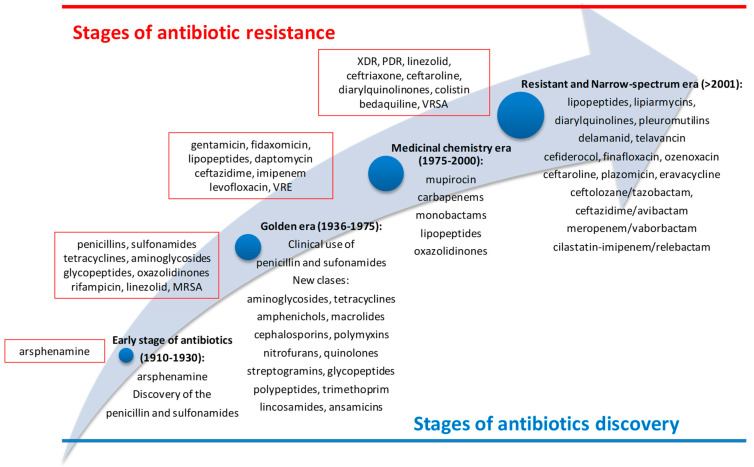
The evolution of antibiotics discovery and their resistance (MRSA—methicillin-resistant *Staphylococcus aureus*, PDR—pan-drug-resistant, VRE—vancomycin-resistant enterococci, VRSA—vancomycin-resistant *Staphylococcus aureus*, XDR—extensively drug-resistant) [2,5,6,8].

The clinical use of antibiotics started at the beginning of the 20th century, when arsphenamine, a toxic organoarsenic compound, was used in syphilis treatment. Until 1938, when penicillin was introduced in therapy, arsphenamine had been the appropriate option for this pathology [1]. Despite the use of arsphenamine, the antibiotherapy era had started in 1936 when sulfonamides (inhibitors of dihydropteroate synthetase) were used for the first time in therapy [2]. During the golden era (1936–1975), many classes of antibiotics with different spectra of activity and mechanisms of action were discovered. This was the most fruitful period in the history of antibiotics with high significance for clinical use (β-lactams—1938, aminoglycosides—1946, tetracyclines—1948, amphenichols—1949, polymyxins—1950, macrolides—1951, nitrofurans—1953, quinolones and trimetoprim—1962, lincosamides and ansamycins—1963, cephalosporins—1964, streptogramins—1965, phosphonates—1971, etc.) (Figure 1). Unfortunately, shortly after their first use in therapy, clinicians discovered a pressing problem, the lack of antibiotic efficacy. This inefficiency of the antimicrobial drug against the growth and multiplication of microorganisms was named antimicrobial resistance (AMR) [3]. Thus, from the beginning of the widespread use of penicillin, its discoverer, Sir Alexander Fleming, warned of the risk of its uncontrolled use leading to AMR. In fact, after only one year of use, the first cases of infections with penicillin resistance, *Staphylococcus aureus*, were observed [4]. In this context, it was necessary to intensify the research in the field, and the medicinal chemistry era (1975–2000) started. The research was conducted to obtain synthetic compounds with broad spectrum (carbapenems and mupirocin—1985, monobactams—1986, oxazolidinones—2000, lipopeptides—2003) [5]. During the last two decades, novel classes of antibiotics have been discovered (pleuromutilins—2007, macrolactones—2011, diarylquinolines—2012, catechol-substituted siderophores—2019) [2,5,6,7]. Simultaneously, other molecules from older classes were introduced in clinical use: cephalosporins (ceftaroline—2010), aminoglycosides (plazomicin—2018), tetracyclines (eravacycline—2018), beta-lactam/beta-lactamase inhibitor (ceftolozane/tazobactam—2014, ceftazidime/avibactam—2015, meropenem/vaborbactam—2017, and cilastatin-imipenem/relebactam—2019). Their activity is predominant against Gram-negative bacteria and they can be used when bacteria is resistant to third-generation cephalosporines or to carbapenems (Figure 1) [7].

Among the factors that led to the emergence of AMR, the following can be listed: (a) biological factors represented mainly by bacterial evolution and genetic mutations; (b) excessive and abusive use of antibiotics; (c) extensive use of antibiotics in agriculture (in animal or fish feed, in water for the prevention of infections or for the treatment of sick animals); (d) increase of the population income (which generates a direct increase in the consumption of antibiotics, and also indirectly as a result of the increase in consumption of contaminated meat); (e) the possibility of travel or transport of consumer goods allows the spread of microorganisms; (f) incomplete information on the phenomenon of AMR, including statistics on the consumption of antibiotics; (g) lack of information released to the public regarding the correct administration of antibiotics and the risks of misuse; (h) lack of adequate measures adopted by authorities (such as infection management, ensuring optimal conditions in health facilities) [3,9,10]. In addition to the above mentioned, there are several factors that discourage drug manufacturers from investing sufficient funds to develop new antibiotics, leading them to focus their research on the drug classes used to treat chronic diseases. Thus, the following could be mentioned: high costs for research and development of new molecules, the long time required for their authorization, the risk that the antibiotic will soon become ineffective, strict legislation and strict price control [10,11].

Therefore, AMR is a major problem for any health system, with a slow but constant evolution [12] and with multiple implications from a medical, social and economic point of view [3]. The direct consequences of AMR on the patient are the aggravation of pathologies, the compromise of the patient’s immune system, the appearance of various complications and even therapeutic ineffectiveness. At the level of the health system, AMR generates an increase in healthcare costs due to prolonged hospitalization, the need for additional monitoring or more expensive medication. AMR can also reduce the chance of successful medical procedures (such as surgery, antitumor chemotherapy or organ transplantation) or therapeutic success in vulnerable patients (such as diabetic, asthmatic, rheumatic patients, etc.). Last but not least, the social system is influenced by AMR by increasing the costs of declining productivity, the onset of disability and increasing patient mortality [3,10,13], with around 4.95 million deaths worldwide [14] among which 30,000 are in Europe [15]. Such severe consequences have been reported in infections with methicillin-resistant *Staphylococcus aureus* (MRSA), multidrug-resistant Gram-negative bacteria (*Enterobacter* spp., *Escherichia coli*, *Klebsiella pneumoniae*, *Pseudomonas aeruginosa*, etc.), but also in tuberculosis, gonorrhea, candidiasis and typhoid fever [3,16].

Given the current epidemiological context dominated by the COVID-19 pandemic, the use of antibiotics for the treatment of bacterial infections associated with coronavirus SARS-CoV-2 [17] should also be considered. Numerous studies have shown an increased prevalence of multidrug-resistant bacterial infections or even fungal infections during the COVID-19 pandemic [13]. Thus, an abusive and excessive consumption of antibiotics was reported, which led to an increased risk of AMR phenomenon, but also of the occurrence of side effects [13,18,19]. During the COVID-19 pandemic, in order for the use of antibiotics in patients infected with the SARS-CoV-2 virus to be rational, the WHO developed a guideline that antibiotics are recommended only in severe cases of COVID-19, in which case therapy should be reevaluated daily. In these cases, antibiotics will be used only on the basis of clinical diagnosis, local epidemiology, and susceptibility data. It is also stated that empirical therapy should not last more than 5–7 days, and it should be confirmed by clinical evaluation and the results of microbiological tests. The WHO notes that the use of antibiotics can also lead to infections with *Clostridium difficile*, while unjustified use leads to AMR and multidrug-resistant bacteria which increase the number of deaths among COVID-19 patients [9].

Considering all these aspects, the aim of this review is to summarize the main mechanisms of bacterial resistance to antibiotics and to discuss potential therapeutic options for infections caused by MDR bacteria.

## 2. Mechanisms of Action and Antibiotic Resistance of Microorganisms

**Figure 2:**
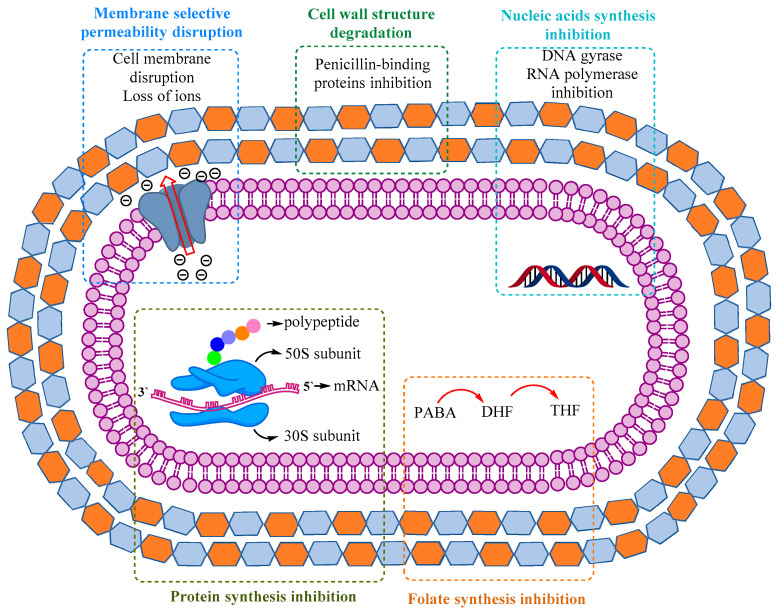
The main mechanisms of action of antibiotics [21,22,23,24].

The mechanisms by which antibiotics act against microorganisms are: inhibition of bacterial cell wall synthesis, alteration of the bacterial cell membrane, inhibition of the protein synthesis, inhibition of nucleic acids’ synthesis (Figure 2).

Figure 2 summarizes the variety of mechanisms responsible for antibiotic activity [20,21] and Table 1 presents the main classes of antibiotics according to their mechanisms of action.

With the introduction of the first antibiotics in therapy, the problem of treating bacterial infections was considered solved, but it soon became clear that bacteria, both Gram-positive and Gram-negative, were able to develop mechanisms of resistance to more and more antibacterial drugs. The increased use of antibiotics has led to the rapid emergence and expansion of the resistance of pathogenic strains. Microorganisms are undergoing more and more mutations, so they manage to survive the action of many antibiotics used currently in therapy. At this rate, bacteria will also acquire resistance to newly approved antibiotics or to those in the research and development phase.

Resistance can be achieved through multiple and complex mechanisms, such as mutations, absorption of exogenous genes, horizontal transfer from other bacterial strains or triggering of a genetic cascade, thus inducing the expression of resistance mechanisms [35]. Microorganisms can also develop resistance through mechanisms that have an impact on the pharmacokinetics and pharmacodynamics of a drug substance, such as: limiting the absorption into the bacterial cell, modifying the drug target site, inactivating of a drug or its active efflux. These mechanisms of resistance may be native characteristics of some species or may be acquired from other microorganisms, and the elucidation of these mechanisms may lead to more effective treatment options for infectious diseases and the development of novel antimicrobial drugs [20].

With the discovery of antibiotics, the continuous pressure of drug-induced selection led to the emergence of microorganisms known as superbugs that are resistant to multiple drugs, such as multidrug-resistant (MDR), extensively drug-resistant (XDR) or pan-drug-resistant (PDR), etc. [20].

Microorganisms that have developed MDR are generally extremely dangerous microbial species with significant pathogenicity, among which are ESKAPE bacteria (*Enterococcus faecium*, methicillin-resistant *Staphylococcus aureus*, *Klebsiella pneumoniae*, *Acinetobacter baumannii*, *Pseudomonas aeruginosa* and *Enterobacter* species), *Mycobacterium tuberculosis*, extended spectrum beta-lactamases (ESBL)-producing bacteria and vancomycin-resistant enterococci (VRE) [36,37,38,39,40].

Antibiotic resistance of microorganisms can be intrinsic or acquired; it is the result of complex factors among which are genes that act directly or indirectly to block the activity of antibiotics. Often, resistant strains can act through several mechanisms, resulting in combinatorial resistance, which ultimately complicates clinical therapy. The existence of precursor or proto-resistance genes has given rise to all highly effective elements that result in the resistance of the microorganism [41,42].

Susceptibility and resistance are assessed in relation to the minimum inhibitory concentration (MIC). If the MIC value for a species is high, bacteria is considered to have intrinsic resistance to that drug. In addition, bacteria can acquire resistance genes from other related microorganisms, and the level of resistance will vary depending on the species and the genes acquired [43,44,45].

The main mechanisms of resistance to antibiotics are (Table 2):

(1) Antibiotic inactivation through the production of specific enzymes: inactivation occurs through the activity of the enzymes produced by bacteria that disorganize and break the specific bonds of the molecule of the antibiotic, making it inefficient. These enzymes are: β-lactamases, ESBL, etc. [46]. These β-lactamases-producing bacteria are a typical source of nosocomial infections, which can vary from uncomplicated urinary tract infections (UTIs) to serious problems like sepsis [47,48,49];

(2) Variations in the permeability of the membrane through which the antibiotic is prevented from entering the cell membrane by regulating porin expression. For example, *P. aeruginosa* strains could become resistant to imipenem due to the loss of OprD porin, necessary for antibiotic penetration through the cell membrane [50,51];

(3) Elimination through efflux pumps, which prevents the accumulation of the antibiotic in the intracellular environment so that it does not reach levels of intracellular concentration that can kill the bacterial cell. Efflux pumps can eliminate a broad range of compounds that can be toxic to the microorganism, which is why they have also been named multidrug efflux pumps. These proteins have an important contribution to the establishment of resistance to many drugs and can occur in several bacteria. Bacterial resistance mediated by efflux pumps is associated with many classes of antibiotics, such as tetracyclines, fluoroquinolones, aminoglycosides, etc. [52,53];

(4) Modification of the target site: the bacteria modify the conformation of the target or prevent the binding of the antibiotic to its site of action. Some resistant bacteria avoid antibiotics through the reprogramming or camouflage of target sites, in order to escape from being recognized by these substances. Thus, even if the intact and active antimicrobial compound is present in the intracellular medium, no further binding or inhibition will take place. For example, in *Staphylococcus aureus* there is a diminished affinity of the antibiotic for penicillin-binding proteins (PBP) due to alteration of the protein-binding site, so that the bacteria continue to grow and to multiply even at large antibiotic concentrations [54]. This mechanism of resistance is also employed during [55,56,57]:

- Modification of PBP, which leads to a decreased affinity of beta-lactam antibiotics (methicillin-resistant *Staphylococcus aureus*, *Streptococcus pneumoniae*, group A *Streptococcus*, *Listeria monocytogenes*, *Neisseria gonorrhoeae*);
- Modifications in the structure of peptidoglycan and in the thickness of the cell wall resulting in diminished activity of vancomycin (vancomycin-resistant *Staphylococcus aureus*, VRSA);
- Alteration of the D-Ala-D-Ala ligase (vancomycin-resistant *Enterococcus faecium* and *Enterococcus faecalis*);
- Modifications of the DNA-gyrase subunits which led to decreased activity of fluoroquinolones: thus, many Gram-negative bacteria have developed resistance to this class of antibiotics;
- Modification of topoisomerase IV subunits that reduce the activity of fluoroquinolones (many Gram-positive bacteria, especially *Staphylococcus aureus* and *Streptococcus pneumoniae*);
- Alteration of the RNA polymerase, leading to a reduction in the activity of rifampicin activity against *Mycobacterium tuberculosis*;
- Modification of 16S ribosomal rRNA or ribosomal proteins: *Mycobacterium* spp.

**Table 2:** Main mechanisms of bacterial resistance to different classes of antibiotics.

Mechanism of Resistance		Classes/Examples	References
Altered target	PBP	*Beta-lactams*: Penicillins, Cephalosporins, Carbapenems, Monobactams	[58,59,60]
	Peptidoglycan biosynthesis (D-Ala-D-Ala ligase)	*Glycopeptides*: Vancomycin, Teicoplanin	[61,62]
	Overproduction of capsular polysaccharide	*Cationic peptides*: Colistin, Polymyxin E	[63,64]
	Lipopolysaccharides from bacterial outer membrane	*Cationic peptides*: Colistin, Polymyxin E	[63,64]
	Ribosomal subunit	*Aminoglycosides*: Amikacin, Gentamicin, Kanamycin, Spectinomycin, Streptomycin, Tobramycin	[65,66]
		*Macrolides*: Erythromycin, Clarithromycin, Azithromycin	[67,68]
		*Tetracyclines*: Tetracycline, Doxycycline, Minocycline, Tigecycline	[69,70]
		*Streptogramins*: Quinupristin and dalfopristin	[30]
		*Oxazolidinones*: Linezolid	[71]
		*Lincosamides*: Clindamycin	[72,73]
	DNA gyrase	*Fluoroquinolones*: Ciprofloxacin, Ofloxacin, Levofloxacin, Sparfloxacin	[72,74]
	RNA polymerase	*Rifamycins*: Rifampin	[72,75]
	Folate inhibitors	*Folate inhibitors*: Trimethoprim Sulfonamides	[76,77]
Efflux pumps	Reduction of antibiotic absorption	*Aminoglycosides*: Amikacin, Gentamicin, Kanamycin, Spectinomycin, Streptomycin, Tobramycin	[65,66]
		*Beta-lactams*: Penicillins, Cephalosporins, Carbapenems, Monobactams	[58,59,60]
		*Tetracyclines*: Tetracycline, Doxycycline, Minocycline, Tigecycline	[69,70]
		*Streptogramines*: Quinupristin and Dalfopristin	[30,72]
		*Oxazolidinones*: Linezolid	[71]
		*Lincosamides*: Clindamycin	[72,73]
		*Fluoroquinolones*: Ciprofloxacin, Ofloxacin, Levofloxacin, Sparfloxacin	[72,74]
		*Folate inhibitors*: Trimethoprim Sulfonamides	[76,77]
		*Macrolides*: Erythromycin, Clarithromycin, Azithromycin	[67,68]
		*Cationic peptides*: Colistin, Polymyxin E	[63,64]
		*Rifamycins*: Rifampicin	[72,75]
Enzymes	Hydrolysis	*Beta-lactams*: Penicillins, Cephalosporins, Carbapenems, Monobactams	[58,59,60]
		*Macrolides*: Erythromycin, Clarithromycin, Azithromycin	[67,68]
	Acetylation	*Amoglycosides*: Amikacin, Gentamicin, Kanamycin, Spectinomycin, Streptomycin, Tobramycin	[65,66]
		*Fluoroquinolones*: Ciprofloxacin, Ofloxacin, Levofloxacin, Sparfloxacin	[72,74]
		*Streptogramines*: Quinupristin and Dalfopristin	[30,72]
	Carbon-Oxygen lyase	*Streptogramines*: Quinupristin and Dalfopristin	[30,72]
	Phosphorylation	*Lincosamides*: Clindamycin	[72,73]
		*Macrolides*: Erythromycin, Clarithromycin, Azithromycin	[67,68]
		*Aminoglycosides*: Amikacin, Gentamicin, Kanamycin, Spectinomycin, Streptomycin, Tobramycin	[65,66]
	Glycosylation	*Macrolides*: Erythromycin, Clarithromycin, Azithromycin	[67,68]
	Nucleotidylation	*Lincosamides*: Clindamycin	[72,73]
		*Aminoglycosides*: Amikacin, Gentamicin, Kanamycin, Spectinomycin, Streptomycin, Tobramycin	[65,66]
	Hydroxylation (under FAD-requiring monooxygenases TetX and TetX2,)	*Tetracyclines*: Tetracycline, Doxycycline, Minocycline, Tigecycline	[69,70,78]

**Figure 3:**
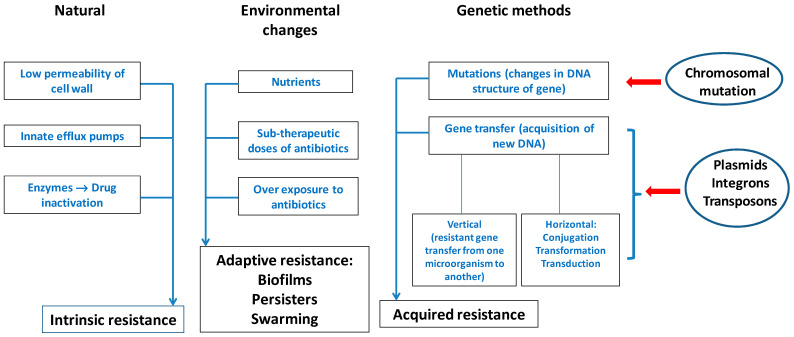
Various elements of bacterial resistance to antibiotics. Adapted from [79], published by *Front* *Microbiol*, 2013 and [80], published by *Environ Sci Pollut Res*, 2019.

Through various mechanisms, microorganisms can develop resistance to antibiotics, which often leads to the ineffectiveness of the antibiotic. Thus, there are two important types of resistance mechanisms: (1) intrinsic/natural resistance; (2) acquired resistance (Figure 3).

### 2.1. Intrinsic Resistance of Microorganisms

The natural resistance of microorganisms to various medicinal agents can be intrinsic (always expressed within the species) or induced (genes occur naturally in bacteria, but are expressed only after exposure to an antibiotic).

Intrinsic resistance is a feature controlled by the bacterial genome and represents a species characteristic. It does not depend on the contact with a specific antibiotic and is not triggered by horizontal gene transfer [81].

Certain bacterial species can acquire tolerance to a drug or class of antibiotics due to their structure and functional properties [82].

The most common mechanisms responsible for the intrinsic resistance are: reduced outer membrane permeability of Gram-negative bacteria and the natural activity of efflux pumps [83].

This characteristic of the species can be called “insensitivity” because it appears in organisms that have never been sensitive to a specific therapeutic agent. This natural insensitivity may be a result of different causes, such as [84,85]:

- Absence of antibiotic affinity for the bacterial target;
- Reduced drug uptake into the bacterial cell;
- Extrusion of the drug by chromosomally encoded active carriers;
- Biosynthesis of specific enzymes able to inactivate the antibiotic.

Due to the structural differences of bacteria, especially regarding membranes (Gram-positive or Gram-negative), there are differences in their types of mechanisms of resistance, as follows: Gram-negative bacteria use all four main mechanisms mentioned above, while in the case of Gram-positive bacteria, due to their lack of the phospholipid outer membrane, they do not have the ability to develop certain types of drug efflux mechanisms [86,87].

Currently, the prevalence of infections caused by Gram-negative bacteria is increasing at a dangerous rate, these infections often being difficult to treat due to the intrinsic resistance of Gram-negative pathogens that greatly reduces therapeutic options [79]. The mechanisms presented are additional to the genetic inheritance which may contribute to an increased level of intrinsic resistance. By combining these elements, intrinsic resistance has been defined more precisely and it is increasingly accepted that this phenomenon is much more complex [50].

As previously shown, all microorganisms have their own elements that contribute to the formation of a phenotype characteristic of developing a certain susceptibility to antibiotics, known as intrinsic resistance “resistoma”. The appearance of mutations between these elements determines that some bacteria are more vulnerable to antibiotics, while for others a greater resistance is achieved. However, the acquisition of a phenotype that is more resistant does not always involve a genetic change, either due to mutation or as a consequence of the acquisition of a resistance gene by horizontal gene transfer. Phenotypic resistance, which cannot be inherited, can be acquired through various processes including the emergence and development of microbial biofilms, adaptation to different reproductive pathways and the development of persistence [81].

In therapeutic practice, it is important to know the intrinsic resistance of a microbe in order to avoid therapies that can be inefficient. For some bacterial pathogens (e.g., *Pseudomonas aeruginosa*, *Mycobacterium tuberculosis*) that are by nature resistant to a large number of antimicrobial substances, there is a limited range of therapeutic options and this further escalates the risk of acquired resistance [83].

Intrinsic bacterial resistance was reported for several microorganisms. Among anaerobic bacteria, this phenomenon was observed for: *Sutterella wadsworthensis*, *Fusobacterium* spp., *Clostridium* spp., *Prevotella* spp., *Bilophila wadsworthia*, *Bacteroides* spp. Their natural resistance against aminoglycosides, many β-lactams, quinolones, metronidazole, imipenem, ampicillin-sulbactam and piperacillin-tazobactam can be explained by various mechanisms, such as: inhibition of aminoglycosides uptake determined by the absence of oxidative metabolism or the incapacity to generate the active form of the drug through anaerobic reduction reaction [88,89,90,91,92].

Gram-positive bacteria can produce broad spectrum β-lactamases, a group of bacterial enzymes that can inactivate by enzymatic hydrolysis even third-generation cephalosporins and aztreonam [93].

The acquisition of genetic elements is considered the main mechanism associated with cephalosporin resistance in *Listeria monocytogenes*. Other AMR mechanisms such as horizontal gene transfer, susceptibility to environmental stressors, biofilm formation, the presence of persistent cells and efflux pumps are related to *L. monocytogenes* resistance to some antibiotics, including fluoroquinolones [94].

The existence of AMR genes in lactic acid bacteria (*Lactobacillus* spp., *Lactococcus* spp., *Leuconostoc* spp. and *Pediococcus* spp.) as well as their capacity to transfer to other microorganisms is one of the mechanisms related to natural resistance to aminoglycosides (gentamicin, kanamycin, streptomycin and neomycin), ciprofloxacin and trimethoprim. Whole-genome sequencing probably allows the identification of all possible genetic determinants of antimicrobial resistance in a microbial genome. Lack of suitable cell wall precursor molecules prevents vancomycin from binding and inhibiting cell wall synthesis [95,96].

The mechanism of AMR in Gram-negative bacteria arises from the expression of antibiotic inactivation enzymes and non-enzymatic pathways from the increase of intrinsic resistance due to chromosomal gene mutations or acquired genetic material that carries resistance genes [97,98]. The presence of chromosomal mutations (rplD, rplV and 23S rRNA), ten macrolide resistance genes (MRG) and efflux pump overexpression determined the resistance of *E. coli* to macrolides [99,100]. *Klebsiella* spp. have shown natural resistance to ampicillin, cephalosporins and carbapenems. These bacteria produce enzymes (beta-lactamases) that inactivate the drug before it can reach the PBPs. The resistance is related to the emergence of the blaSCO-1 gene, which mediates the production of class A carbenicillinase-like enzymes, mediated by plasmids of unknown origin, capable of hydrolyzing not only penicillins but also, to a lesser extent, cephalosporins and carbapenems [101,102].

The intrinsic mechanisms underlying AMR predominate through the natural genes found in the host chromosome, such as the multiresistant efflux systems of Gram-negative bacteria being involved in rendering *Serratia marcescens* resistant to ampicillin, macrolides and first-generation cephalosporins [103,104,105]. *Stenotrophomonas maltophilia* is able to produce enzymes (aminoglycoside acetyltransferases, beta-lactamases) that inactivate the antibiotic before it reaches its target. Other mechanisms involve efflux pumps. In addition, these strains may develop multidrug resistance [106,107].

In the case of *Acinetobacter* spp., the microbial genes that cause AMR overexpress efflux pumps or synthetize β-lactamases and the species are characterized by low membrane permeability; within the genus, over 210 β-lactamases have been identified. These enzymes are serine hydrolases of class D according to the Ambler classification of β-lactamases (named oxacylinase-OXA-enzymes). *Acinetobacter* spp. possess natural resistance to ampicillin, oxacillin and glycopeptides [108,109,110,111].

For *Enterococcus* spp., inhibition of aminoglycoside uptake is determined by the absence of oxidative metabolism. Their resistance to all cephalosporins is linked to the lack of PBPs that effectively bind and are inhibited by these beta-lactam antibiotics [112]. Resistance to lincosamides in *Enterococcus* spp. is due to the plasmids that carry the antimicrobial resistance genes [113,114].

For *Pseudomonas aeruginosa*, reduced uptake resulting in lower intracellular concentrations explains the resistance to sulfonamides, trimethoprim, ampicillin, first- and second-generation cephalosporins, tetracycline, and chloramphenicol [113,114].

Interest in intrinsic resistance genes has increased greatly in recent times, as these genetic means not only can provide attractive therapeutic targets for new drugs that enhance the effectiveness of existing antibiotics, but could also predict the future of resistant pathogens.

A representative example of intrinsic antibiotic resistance is given by some MDR Gram-negative bacteria unsusceptible to many classes of antibiotics that are clinically effective in infections produced by Gram-positive bacteria. In Gram-negative bacteria, this phenome is caused by the resistance of the cell membrane to the penetration of a large number of molecules because MDR bacteria efflux pumps efficiently diminish the intracellular concentration of the given drug [101,107,112].

### 2.2. Acquired Resistance of Microorganisms

Acquired resistance happens when pathogens become less sensitive to antibiotics by which they were previously easily affected. This behavior is different from intrinsic resistance as the genes or the mutations responsible for resistance were not initially present [115].

Pathogens, as well as commensal bacteria, are often homologous and are carriers of transferable genetic elements [116].

Complex molecular mechanisms lead to the spread of microbial resistance, such as [117]:

- Genetic transfer mechanisms: conjugation, transformation, transduction;
- Mobile genetic elements (MGEs).

#### 2.2.1. Genetic Transfer: Conjugation, Transformation, Transduction

AMR is transmitted through genetic material transfer that can be “vertical” when the descendants receive antibiotic-resistant genes or “horizontal” when microorganisms (bacteria and viruses) interchange fragments of genetic material [118].

About 70 years ago, the introduction of experimental microbial genetics made possible the horizontal gene transfer (HGT), thus identifying the growing problem of the evolution of antibiotic resistance of pathogenic bacteria [119]. Research in the field of bacterial genetics has shown that horizontal gene transfer is responsible for some of the genetic variations that cause antibiotic resistance [120].

**Figure 4:**
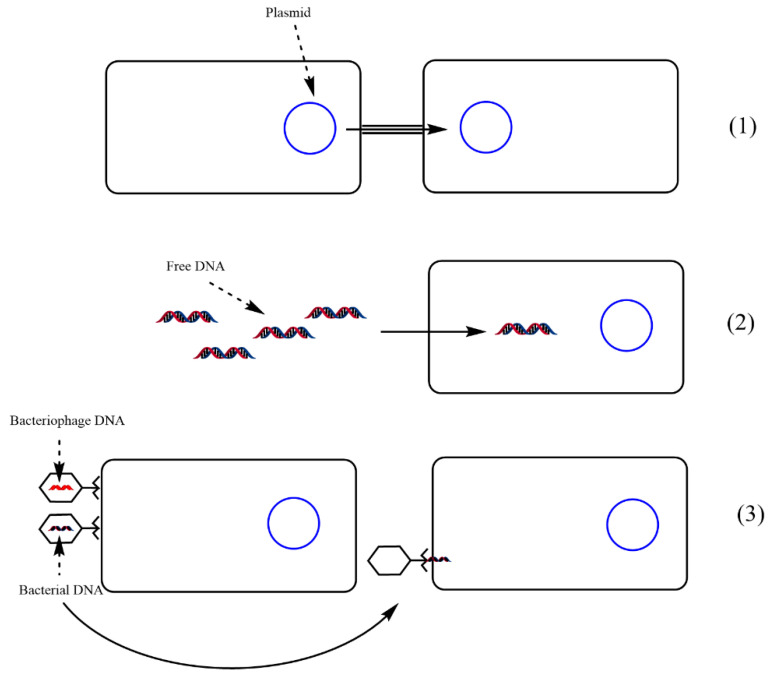
Genetic transfer in AMR—(**1**) conjugation, transfer of genes from one bacterial cell to another that requires cell-to-cell contact, (**2**) transformation—uptake of free DNA from the environment, (**3**) transfer of plasmid genes from one cell to another by viruses. Adapted from [128], published by *Pharmaceuticals and Personal Care Products: Waste Management and Treatment Technology*, 2019.

HGT occurs through three main mechanisms: (1) conjugation, (2) transformation and (3) transduction (Figure 4). The succession of these stages is essential for a better understanding of the molecular mechanism of AMR:

(1) Conjugation is the most common mechanism responsible for the transfer of resistance genes, being a process carried out in several stages and which requires close cellular contact between the donor and the recipient cell. In this type of gene transfer, a fertility factor F is present in the donor cell, which is an autonomous DNA molecule [121];

(2) Transformation involves the absorption of cloned DNA, which is released into the environment as a result of cell lysis and is incorporated into the host cell by integration into the genome or by recirculation of the DNA molecule (through plasmids) [121,122];

(3) Transduction is the result of some bacteriophages (viruses) infecting a bacteria and incorporating a part of the viral genome into the host cell or transferring particular genes into the cell [123,124]. It has been documented that antibiotic-resistant genes are mobilized by bacteriophages for different bacterial species [125], but the role of bacteriophages in the mechanism of antimicrobial-resistant genes transfer is not fully elucidated and is still controversial [126,127,128].

In 2017, WHO developed a list (Table 3) of antibiotic-resistant microorganisms responsible for severe diseases, in order to increase the global awareness of the AMR phenomenon.

#### 2.2.2. MGEs

MGEs play a critical role in the evolution and persistence of AMR. MGEs consist of different genes and subelements which allow a large array of interactions with other genetic elements that promote their adaptability and endurance, such as: plasmids, integrons, transposons (Tn), insertion sequences (IS) and genomic islands; these are able to activate resistant genes from various species of bacteria, animal hosts and the environment [129]. These MGEs are among the most important factors in the evolution of antibiotic resistance due to the lateral gene transfer (LGT), a significant ability of bacteria that allows them to share genetic resources [130,131].

- Plasmids are small DNA molecules that can reproduce separately from the host chromosome and are different from the bacterial chromosome because they do not carry vital genes, but genes that can be beneficial for the host cell [132]. The cell to cell transfer of plasmids occurs through conjugation and transformation, and the spread of resistant genes is primarily caused by plasmids that confer resistance to some antibiotics, such as β-lactams, carbapenems and colistin [133,134].
- Integrons are genetic elements that can capture and rearrange exogenous DNA and incorporate it into an individual genomic unit. The structure of these elements is defined by the expression of an integrase gene (intI) and a recombination site (attI) [135]. Integron mobility is of major importance because they are associated with Tn and plasmids and also play an essential part in the dissemination and spread of AMR [131].
- Transposable elements (TEs) are DNA segments that are able to be mobilized from one site to another; they are inserted into DNA, are not separated and cannot replicate on their own. Chromosomes, plasmids, viral genomes and other DNA molecules can be included among the host molecules for TE. Bacteria possess two main types of TE: IS and Tn.

### 2.3. Resistance versus Persistence and Tolerance to Antibiotics

Taking into consideration the increasing prevalence of antibiotic resistance of microorganisms, it is very important to distinguish between the concepts of resistance, persistence and tolerance to antibiotics. Bacteria resistant to a particular antimicrobial agent will normally cause transmission to descendants of the species, unless additional mutations have occurred in the meantime.

The subpopulation of cells that is able to withstand radical antibiotic treatment without being resistant constitutes the bacterial persister cells.

Over the last two decades, there have been numerous concerns about defining, evaluating and understanding bacterial persistence and establishing its relationship with antibiotic resistance, heteroresistance or tolerance. Microbial persistence to antibiotics is not just an instance of unicellular non-genetic heterogeneity but can also play a substantial role in the failure of antibiotherapy [136].

The persistence of microorganisms to various anti-infective agents is a characteristic of bacterial cells that are not sensitive to the drug and do not possess resistance genes, due to the stationary phase of the microorganism life cycle. Most antimicrobial agents have no effect on cells that do not grow and divide actively [20,137,138].

When bacterial cells are exposed to an antimicrobial agent, there are two possible situations:

1. Cells that are resistant to the antimicrobial agent. The non-resistant cells are killed by the antimicrobial agent, and only the resistant cells will remain and grow.
2. Persister cells (latent, non-resistant) may be present. Sensitive cells are killed, leaving only persister cells. When persister cells develop, the cells that are not in an inactive state will still be susceptible to the antimicrobial agent.

**Figure 5:**
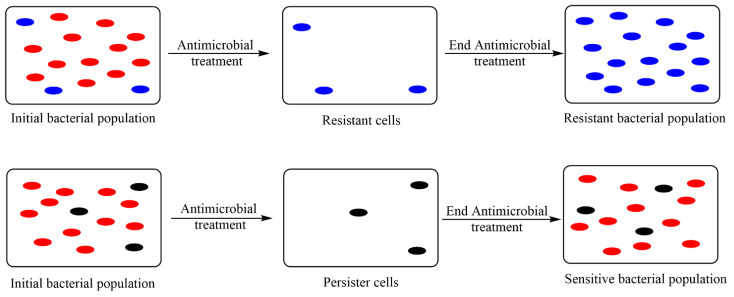
Resistance vs. persistence. Adapted from [20], published by *AIMS Microbiol*, 2018.

The differences between resistant and persister cells are shown in Figure 5.

Antibiotic tolerance among bacteria is the ability to survive longer treatments with bactericidal antibiotics and can result from mutations caused by the evolution of bacterial species, but also from general environmental conditions, which slow down the growth of the microorganisms. As with resistance, persistence and tolerance were first observed shortly after penicillin was introduced [139].

A high degree of persistence or tolerance to antibiotics leads to an increased number of viable bacterial cells during antibiotic treatment, which leads to a higher probability of mutations that confer antibiotic resistance.

Stress responses also play a significant part in the occurrence of persister cells and can cause a temporal surge in cell mutation rates [140,141,142]. Thus, elevated levels of persistence and mutation rates can operate synergistically under stress conditions and increase the probability of resistance mutations [143]. The consequences of the responses to bacterial stress, as a main determinant of the link between resistance and persistence, are illustrated by the example of the emergence of persistent infections caused by *Mycobacterium* strains due to high levels of oxidative stress [144].

Studies of *Saccharomyces cerevisiae* have shown an increased level of modified DNA, which indicates a high mutation rate [145]. Recent research has suggested that heterogeneity in the expression of the AcrAB-TolC multidrug efflux pump may generate a subpopulation that not only has transient resistance to multiple drugs, but is also characterized by slowdown growth and a reduced expression of the MutS DNA repair enzyme, leading to a higher rate of spontaneous mutation [146].

The emergence of antibiotic tolerance allows bacteria with an improved genetic background to withstand the action of broad-spectrum antibiotic treatments [147]. Activating bacterial metabolism has been suggested to counteract antibiotic tolerance by converting tolerant cells to sensitive ones, using exogenous metabolites such as nucleotides, amino acids and tricarboxylic acid cycle metabolites [148]. A better understanding of the in vivo mechanisms involved in antibiotic tolerance may in particular contribute to the targeting of potential steps of bacterial metabolism. Antibiotic tolerance has been shown to be closely associated with slow or reduced bacterial metabolism [139]. So, an increase in metabolism may bring back the susceptibility of tolerant bacteria to antibiotics and adding specific exogenous metabolites can enhance the metabolic status.

Two representative mechanisms for tolerance have been identified, namely “slow-growing tolerance” and “delayed tolerance”, both related to metabolism alterations [149].

In various strategies for the development of new drugs, metabolic activity offers a path to potentiate the action of antibiotics, based on the observation that the metabolism of a microorganism is closely related to the environment, especially to nutrients. Thus, changes in bacterial metabolism can restore sensitivity to antibiotics with the addition of exogenous metabolites that interfere with the bacterial growth cycle [83].

Additionally, there are studies that surprisingly indicate that drug-tolerant and persister cell species are involved in tumor recurrence [150]. Several pieces of evidence indicate that this phenotypic variability acts as an important factor in the development of resistance to therapy [151]. This parallel between drug resistance of neoplastic cells and infections shows that research on the phenomenon of persistence may contribute to obtaining favorable results in the treatment of cancer [152]. In the mechanism of bacterial persistence, inhibition of lipid hydroperoxidase GPX4, needed for the survival of these species, leads to cellular apoptosis, and therefore hinders the acquisition of drug resistance by neoplastic cells [153]. Both microbiologists and clinicians need to understand, detect and target tolerance and persistence; this should ultimately lead to a decrease in the therapy failure rates regarding infectious diseases and cancers [154].

Therefore, it is considered that tolerant and persister cells are responsible for recurrent bacterial infections with major impact in the medical and industrial field, these phenotypes being related to antibiotic resistance [136,155].

### 2.4. Microbial Biofilms

Biofilms are multicellular assemblages of microorganisms protected by an extracellular matrix that enables microorganisms to grow under various conditions [156].

Through the microscopic investigation of natural ecosystems, it has been proved that more than 99.9% of bacteria grow in biofilms [157]. Using the same microscopic techniques, microbial communities in the form of biofilm have been discovered in chronic infections and on various surfaces [158,159].

An association of microorganisms in the form of biofilm can consist of a single bacterial species, but almost always the microbial biofilm is composed of different bacterial species, and also fungi, protozoa, other microorganisms, debris and degradation products etc. [160].

For example, in dentistry, dental plaque biofilms comprise several hundreds of bacterial species [161,162]:

(1) Gram-positive bacteria ferment carbohydrates especially in a diet high in carbohydrates and thus will develop in the dental biofilm, causing demineralization of teeth and tooth decay, further leading to inflammation and even necrosis in the dental pulp and periapical region;
(2) Gram-negative germs predominate in the supragingival and subgingival biofilms, where anaerobic proteolytic bacteria can also be found, colonizing and causing inflammation of gums and decomposition of periodontal fibers and bones and possibly tooth loss, leading to gingivitis, chronic periodontitis or aggressive peri-implantitis. In addition, bacteria in the dental biofilm can cause systemic problems such as bacteremia by spreading to other parts of the body.

**Figure 6:**
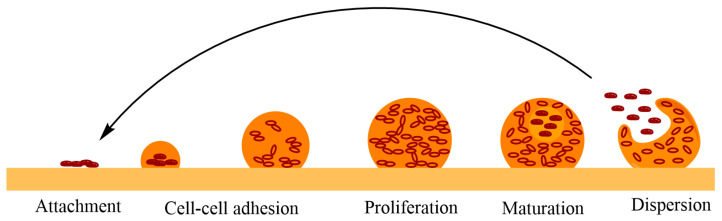
Stages of microbial biofilm formation. Adapted from [163], published by *Antimicrobial Resist Infect Control*, 2019 and [164], published by *Front Chem*, 2019.

Biofilms are surrounded by saccharide molecular chains called “extracellular polymeric substances” (EPS). The cells produce EPS and are aggregated by these chains, granting them the possibility to develop resilient, stable and complex microbial communities that are three-dimensional (Figure 6). Biofilms are the size of a few layers of cells or even a few centimeters in thickness, depending on environmental conditions [163,164].

As early as 1982, a report highlighted a large number of *Staphylococcus aureus* cells incorporated into a biofilm, which caused a systemic infection [165].

Biofilm formation is a way in which microorganisms protect themselves from other microorganisms, and which allows them to survive in harsh conditions and offers the possibility of spreading to other surfaces.

Bacterial biofilms can be present on contaminated surfaces and can play different roles in nature, medicine and industry, sometimes beneficial, sometimes harmful. Microbial biofilms can have health benefits when considered normal components of the microbiome and can be critical to the efficiency of some industrial processes, such as wastewater treatment. However, biofilms can often cause major problems as they could be a reason for chronic infections and could contaminate various surfaces and the environment in industry, affecting the technological process, etc. [166,167].

The eradication of biofilm is very difficult because, as some researchers have concluded, the toughness of the microbial biofilm is due to tolerant and persister cells that can survive antibiotic treatment [158,168].

Approximately 80% of human infections, mainly chronic infections (such as cystic fibrosis, endocarditis and osteomyelitis) are caused by bacterial biofilms. The ability of biofilm to withstand most antibiotics that are available has posed a dangerous threat to various forms of life [163].

The key factor in the antibiotic resistance of microbial communities, in the form of biofilms, is the multicellular nature of the biofilm, the major cause of resistance mechanisms. There are numerous studies that have demonstrated the mechanism of biofilm formation: bacterial cells are held together by EPS, leading to associations of multiple cells that create the heterogeneous environment within the biofilm [169,170]. If the development of the multicellular structure of the biofilm can be disrupted, the effectiveness of antibiotics as well as the defense of the host organism could be increased, which can lead to the eradication of a persistent infection [170,171,172,173,174].

Recent studies have highlighted that peptide dendrimers TNS18 and G3KL have shown promising activity in eradicating MDR bacteria (such as *Staphylococcus aureus*, *Pseudomonas aeruginosa* and *Acinetobacter baumannii*, etc.) biofilms [175,176]. Peptide dendrimers are able to damage the thickness and morphological structure of the biofilm in a dose-dependent manner, leading to complete dispersion of the biofilm [15]. Due to their branched structure, some peptide and glycopeptide dendrimers have remarkable stability, preventing the activity of proteases [177,178,179]. Thus, dendrimers encoded G3KL and TNS18 were studied. They are polycationic hydrophobic polymers without carbohydrate ramifications, which intensely inhibit the biofilm under the MIC by interacting with the bacterial cell membrane [180,181].

## 3. Types of Resistance and Active Medication

The development of AMR is still a major global concern, being one of the most important challenges of the 21st century [182]. Public health assessment in the context of antimicrobial resistance is necessary for the estimation of clinical reference points (mainly morbidity and mortality) and economic indicators (direct costs, resource use and medicine expenditures).

In order to limit AMR, different strategies have been suggested, an example being the diversity in the use of antimicrobial drugs and the use of combinations: the administration of two or more drugs, either alternatively or simultaneously, preferably using drugs with different mechanisms of action [183,184].

Microbial species, over time, since the introduction of antibiotics into therapy, have developed more or less specific resistance mechanisms. Thus, in the following are some examples of resistant microbial species to different antibiotics, the mechanisms of resistance, the infection developed and the antibiotics indicated for treating these infections:

- Methicillin-resistant *Staphylococcus aureus*;
- Vancomycin-resistant *Staphylococcus aureus*;
- Antibiotic-resistant *Streptococcus pneumoniae*;
- Vancomycin-resistant *Enterococcus* spp.;
- Antibiotic-resistant *Clostridium difficile*;
- Carbapenem-resistant *Enterobacteriaceae* spp.;
- Multidrug-resistant *Pseudomonas aeruginosa* [183,184].

### 3.1. Methicillin-Resistant Staphylococcus aureus

Worldwide, the second leading cause of death is represented by infectious diseases, *Staphylococcus aureus* being a human pathogenic bacterium commonly involved in various infections. *Staphylococcus aureus* belongs to the normal bacterial flora of the upper respiratory tract that can colonize other areas, leading to major dermatology or cardiac infections, bacteremia, pneumonia, osteomyelitis, etc. *S. aureus* is also the major cause of postoperative surgical site infection (SSI) and is an important cause of bloodstream infection (BSI) [178].

In recent decades, because of the development of the AMR phenomenon but also due to the abuse of antibiotics, drug resistance of *S. aureus* has steadily increased, the global rate of MRSA infections has grown and clinical anti-infective treatment for MRSA has become increasingly challenging [180]. In approximately 25–30% of healthy individuals, there is a colonization with *S. aureus* on the skin and nasopharyngeal membranes, without having a pathogenic profile and not causing infections in an immunocompetent patient [181,185]. Still, *S. aureus* can generate a wide range of infections; some are benign infections of the skin or soft tissues and others can endanger the patient’s life because they cause serious systemic diseases. Therefore, the emergence of MRSA is a major public health problem [186,187].

MRSA can cause a wide range of infections: endocarditis, meningitis, skin and soft tissue infections (cellulite, abscess), osteomyelitis, pneumonia, UTIs that seldom require incision/drainage or debridement. MRSA has developed resistance to common antibiotics: beta-lactams, including amoxicillin, methicillin, nafcillin, penicillin, oxacillin and cephalosporins. Outpatient oral antibiotic therapy for MRSA infections with a variable duration of 7–10 days [188,189] includes clindamycin, delafloxacin, doxycycline, linezolid, minocycline, omadacycline, tedizolid, trimethoprim-sulfamethoxazole. Antibiotics for injection, intravenously or intramuscularly, are needed for more complicated infections: vancomycin, linezolid or daptomycin [190,191,192] or teicoplanin [193]. Moreover, the association daptomycin-ceftaroline showed promising outcomes in refractory MRSA bacteremia [194].

### 3.2. Vancomycin-Resistant Staphylococcus aureus (VRSA)

Vancomycin is an antibiotic isolated from *Streptomyces orientalis* cultures since 1957. Its antimicrobial spectrum includes various Gram-positive and Gram-negative bacteria (*Staphylococcus* spp., *Enterococcus* spp., *Streptococcus* spp., *Pneumococcus* spp., *Listeria* spp., *Corynebacterium* spp. and *Clostridium* spp.). It can also be prescribed for the treatment of MRSA infections and to patients with allergies to beta-lactams [195,196,197].

During the 1990s, some strains of *S. aureus* intermediate-resistant to vancomycin (VISA) were identified (MIC ≤ 8 µg/mL). The molecular basis of VISA resistance is polygenic and includes gradual mutations in genes that encode molecules that mostly concern bacterial cell wall biosynthesis. In 1997, the first cases of VISA were officially announced in Japan [198].

VRSA isolates (MIC ≥ 16 µg/mL) have also been identified and have been reported since 2002 [199,200]. The vanA gene (one of the most prevalent genetic determinants associated with VRSA) [194] and operon existing on a plasmid are responsible for conferring resistance in *S. aureus* strains, but the total number of infections produced by this microorganism is quite low. By comparison, the number of VISA infections is rather high, and the molecular mechanisms of resistance are not very well-determined. VISA infections are usually associated with persistent infections, failure of vancomycin treatment and poor clinical outcomes [201,202].

VRSA and VISA could cause dermatological infections, lung infections (pneumonia), infections of the brain (meningitis) and of the urinary tract. Characteristics of the VISA phenotype are an increase in the thickness of the cell wall [203]; reduction of peptidoglycan crosslinking and decrease of the autolytic activity of enzymes from the bacterial cell wall [204,205] and dysfunctions of the accessory gene regulator system and changes in growth factors [206].

Treatment options for VRSA infections may include [207] daptomycin (associated with ceftaroline), telavancin, ceftaroline, tedizolid, linezolid, oritavancin [203,207].

### 3.3. Antibiotic-Resistant Streptococcus pneumoniae

Phenotypic expression of *S. pneumoniae* resistance to beta-lactams occurs as a result of a genetic structural change in the proteins that bind these antibiotics [208]. Macrolides resistance is quite significant, so it is estimated to be between 20% and 40% for strains isolated from *S. pneumoniae*. Mechanisms of resistance to macrolide are represented by: alteration of the target ribosomal site, alteration of the antibiotic transport pathway and degradation of the antibiotic [209]. Approximately 22% of *S. pneumoniae* isolates are resistant to clindamycin. Similar to macrolide resistance, AMR to clindamycin involves a change in the antibiotic’s target site [210]. The prevalence of fluoroquinolone (FQ) resistance is generally low, although there are studies showing an increase in AMR to FQ. Resistance of *S. pneumoniae* to FQ occurs through the following mechanisms: bacterial gene mutations, the acquisition of genes encoded by plasmids and increased efflux mechanism [211,212,213]. The AMR of *S. pneumoniae* also increased for the tetracycline class, and the main mechanism is mediated by 2 genes that confer ribosomal protection [214]. The prevalence of resistance to trimethoprim-sulfamethoxazole (TMP-SMX) is approximately 35%. As with FQ, resistance to TMP-SMX is a consequence of mutations in the bacterial genome [215].

The most important infections caused by *S. pneumoniae* are pneumonia, ear, nose, throat infections, hematological infections and meningitis [216,217]. Penicillin-resistant strains are also frequently resistant to many other classes of antibiotics. Thus, resistance to beta-lactams, macrolides, lincosamides, tetracyclines, trimethoprim-sulfamethoxazole and fluoroquinolones was highlighted [218].

Medication includes cephalosporins, such as: ceftriaxone, cefotaxime, ceftaroline [219] and antibiotics of different classes: vancomycin, fluoroquinolone (moxifloxacin, levofloxacin), high doses of beta-lactams (amoxicillin, amoxicillin-clavulanate) and macrolides (azithromycin, clarithromycin, erythromycin). Macrolides that have poor penetration into the cerebrospinal fluid are excluded because they are ineffective in the treatment of meningitis [220,221].

### 3.4. Vancomycin–Resistant Enterococcus

*Enterococcus* species have developed mechanisms of resistance to several antimicrobial agents. Intrinsic resistance mechanisms include low-affinity antibiotic-binding proteins and production of beta-lactamases. The mechanisms of resistance to vancomycin are due to changes in peptidoglycan cell wall structure.

The main mechanism of action of vancomycin is represented by the inhibition of peptidoglycan synthesis by binding to the terminal units of the D-Ala-D-Ala amino acid chain. Modification of this terminal chain decreases the antibiotic affinity for this target, causing the resistance of the microorganism to the action of vancomycin. This behavior is encoded by genotypes that are identified as VanA to VanG. The most frequent are the VanA and VanB genotypes, followed by the VanD and VanC chromosomal phenotypes. Bacterial strains of VRE have a slightly different resistance to aminoglycosides [222,223].

VRE infections often occur in the hospital environment and can be easily transmitted from person to person. VRE infections can be part of polymicrobial infections. Types of VRE infection include meningitis, urinary tract infections, circulatory system infections, endocarditis, post-surgery and catheter-related infections [224,225].

Treatment for VRE infections should be initiated based on the clinical history or failure of previous antibiotic regimens [224,225]. First-line treatments in VRE infections associated with *E. faecalis* [226,227,228,229] are: ampicillin +/− sulbactam, streptomycin, gentamicin, ceftriaxone. Linezolid [230] and daptomycin could also be used [231] and the combination of some of them is considered optimal.

### 3.5. Antibiotic-Resistant Clostridium difficile (ARCD)

Worldwide, *Clostridium difficile* (syn. *Clostridioides difficile*) infections (CDI) became the most common nosocomial intestinal infection, posing a serious threat to Europe and the United States [1,232]. In the early 2000s, ribotype 027 led to higher morbidity, mortality and increased medical costs [233,234].

ARCD infection is caused by the exposure of the normal intestinal microbiome to antibiotics that are inefficient against *C. difficile*, disrupting the former and allowing the latter’s proliferation. Thus, there are many antibiotics associated with an increased risk of developing *Clostridium difficile* infections: ampicillin, amoxicillin, cephalosporins, aminoglycosides, lincomycin, clindamycin and fluoroquinolones (ciprofloxacin, moxifloxacin, levofloxacin) [235,236]. The use of antibiotics is the most common risk factor for the emergence of ARCD, but *C. difficile* infection can recur because this microorganism can survive antimicrobial therapy upon cessation of therapy. It is also known that *C. difficile* is resistant to many antibiotics frequently used for treating bacterial infections [237]. Statistical evaluations based on many studies has showed AMR as follows: clindamycin and erythromycin (10–100)%, cephalosporins and fluoroquinolones (50%) [238]. Another study has showed that the great majority (more than 79%) of the strains tested with second-generation cephalosporins or fluoroquinolones developed resistance very frequently. The third-generation cephalosporins and broad-spectrum fluoroquinolones lead to AMR less often, for a third of the tested strains [238].

Antibiotic resistance of *C. difficile* causes the occurrence or the recurrence of infection. It has a decisive part in the emergence of new types of strains, often causing suboptimal results that can conduce to inefficient treatment. Relapse after *C. difficile* infection, known as (rCDI) affects ~25% of patients after completion of standard therapy and is associated with substantial health care costs. To prevent rCDI in patients at risk, bezlotoxumab, the first monoclonal antibody indicated against toxin C, was developed [239,240].

*C. difficile* cause life-threatening diarrhea and colitis in patients with recent antibiotic therapy. The infection can spread due to poor hygiene in the hospital environment, non-compliance with these conditions or spread from person to person. Infection requires isolating infected patients and stopping treatment with antibiotics that have caused CDI [241,242]. Resistance is due to metabolism alteration, genetic mutation and biofilm formation. Treatment options include vancomycin, fidaxomicin, metronidazole, minocycline, azithromycin, clarithromycin [243,244] and bezlotoxumab monoclonal antibody. The transplantation of fecal microbiota is necessary for recurrent *C. difficile* infections [245,246]. Warnings were stated regarding the practice of bacterial transplantation in 2019 [247].

### 3.6. Carbapenem-Resistant Enterobacteriaceae (CRE)

Adaptation of microorganisms to various antibiotics has generated increasingly effective defense mechanisms, so that the resistance genes encoding this information have led to the occurrence of highly resistant pathogens.

Gram-negative bacteria have expanded resistance. This is determined on the one hand by the multiple structural adaptations and on the other hand by the antibiotic degradation enzymes (e.g., ESBL, AmpC cephalosporinases and carbapenemases etc.) [248].

Carbapenamase enzymes are classified into class A, B and D [249]:

- Class A includes the most common carbapenemase—*Klebsiella pneumoniae* carbapenemase and imipenem-beta-lactamase;
- Class B includes metallo-beta-lactamase such as New Delhi metallo-lactamase. These are located on plasmid vectors and other transport elements. Because of a large variability (15–70%) these enzymes can evade molecular testing;
- Class D comprises OXA enzymes (carbapenemase hydrolyzing oxacillin), resembling ESBL genes, making it difficult to separate the two by molecular testing methods [250].

**Figure 7:**
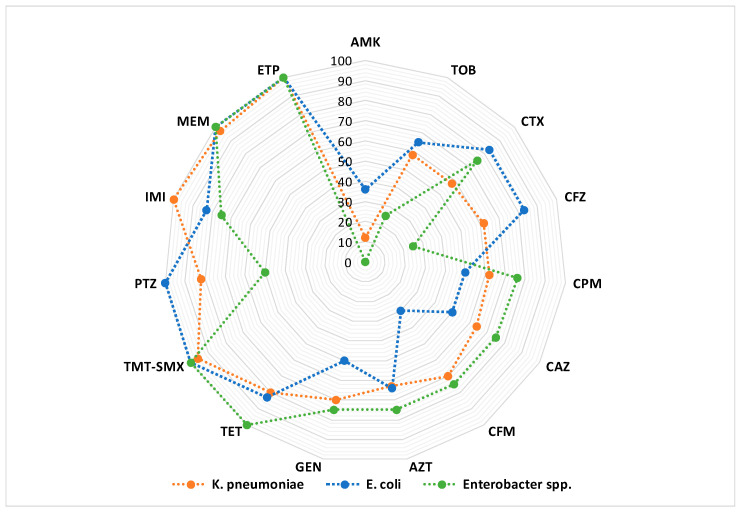
Rate of antimicrobial resistance of *K. pneumoniae*, *E. coli* and *Enterobacter* spp. isolates to different antibiotics (AMK—amikacin, AZT—aztreonam, CAZ—ceftazidime, CFM—cefixime, CFZ—cefazolin, CPM—cefepime, CTX—cefotaxime, ETP—ertapenem, GEN—gentamicin, IMI—imipenem, MEM—meropenem, PTZ—piperacillin-tazobactam, TET—tetracycline, TMT-SMX—trimethoprim-sulfamethoxazole, TOB—tobramycin). Adapted from [255], published by *Infect Drug Resist*, 2020.

*Enterobacteriaceae* spp. are saprophytic microorganisms, which under certain conditions become pathogenic. The high mortality rate in case of serious infections with CRE microbial species explains the high concern for this type of bacterial resistance [251]. Among the carbapenem-resistant microorganisms, the following are noted: carbapenem-resistant *Klebsiella pneumoniae* (CRKP), *Escherichia coli* and *Enterobacter cloacae* (Figure 7). Thus, high mortality rates, between 30% and 75%, have been reported in patients with severe CRE infections [252]. Mortality over 50% has been reported in patients with CRE blood infections [253], and a mortality of 27% in patients with pneumonia or blood infection caused by carbapenem-resistant *K. pneumoniae* [254]. This high mortality associated with CRE is generally attributed to the lack of adequate treatment options and delayed initiation of effective therapy [251].

Moreover, besides the Enterobacteriaceae family, there are other Gram-negative bacteria resistant to carbapenems with clinical relevance such as: *Pseudomonas aeruginosa*, *Acinetobacter baumannii* and, more recently, *Stenotrophomonas maltophilia* [256].

Thus, several elements that define the threat of carbapenem-resistant Gram-negative pathogens can be listed: (i) the increasing prevalence of these pathogens worldwide since the beginning of the century [257]; (ii) lack of other safe and effective therapeutic agents after decreased efficacy of carbapenems due to the occurrence of AMR [258]; (iii) high mortality rate associated with infections with carbapenem-resistant Gram-negative bacteria [259].

Infections caused by CRE include lung infections, blood infections, UTIs, abdominal, febrile neutropenia, upper respiratory tract, surgical wound infections and meningitis. CRE infections tend to be nosocomial, particularly in patients with catheters or associated with various medical devices (endoscopes, duodenoscopes). The main resistance mechanism is the production of carbapenemases. blaKPC and blaNDM are the most frequent carbapenemase-encoding genes in CRKP and CREC (carbapenem-resistant *E. coli*) [260,261,262,263]. Recently, several resistance factors have been reported, even more than 2 carbapenemases in a single strain. The importance of these new elements of resistance, transported mainly by transmissible plasmids, is highlighted [264]. In addition, the production of ESBL and/or AmpC enzymes in combination with mutations in membrane proteins (OmpK35, OmpK36), and overexpression of efflux pumps, are important for the occurrence of the carbapenem resistance [265,266].

Combinations of antibiotics from different classes are recommended for treatment: ceftazidime/aztreonam-avibactam, ceftolozane-tazobactam, meropenem-vaborbactam, imipenem-cilastatin, relebactam, plazomicin, eravacycline and cefiderocol, phosphomycin, minocycline, tigecycline [255,256].

### 3.7. MDR Pseudomonas aeruginosa

**Figure 8:**
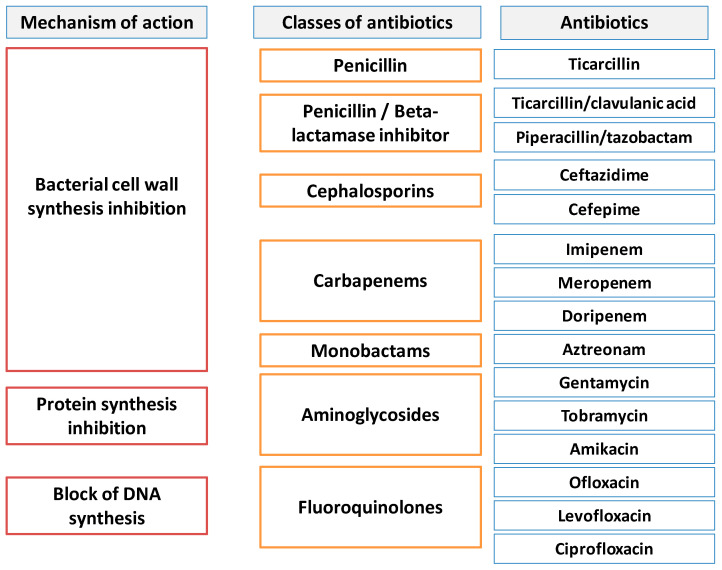
Antibiotics active against *P. aeruginosa*. Adapted from [254], published by *Pathogens*, 2021.

*Pseudomonas aeruginosa* is a saprophytic microorganism belonging to the normal intestinal flora that can become a dangerous pathogen. Nosocomial infections caused by this microorganism are various: gastrointestinal infections, urinary tract infections and septicemia and they are difficult to treat because of a limited number of active antibiotics (Figure 8). Thus, in addition to its intrinsic resistance for β-lactam antibiotics, these bacteria can become resistant to several classes of antibiotics (e.g., aminoglycosides, fluoroquinolones etc.).

**Figure 9:**
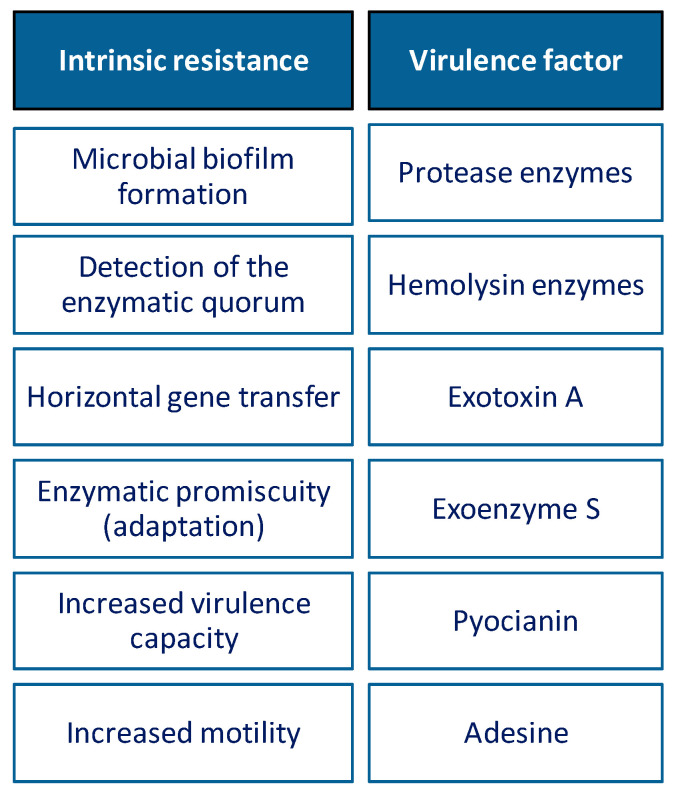
Resistance of *P. aeruginosa* and virulence factors causing extreme pathogenicity. This figure was based on the information provided in [270,271].

In their evolution, these microorganisms have used multiple mechanisms to maintain their genomic plasticity, biofilm formation, enzymatic quorum, horizontal gene transfer and enzymatic adaptation (chromosomal β-lactamase), being the main mechanisms of AMR [267]. Resistance to *P. aeruginosa* is often multimodal, leading to limited antibiotic efficacy in infections caused by this microorganism. These mechanisms could exist at the same time, and could generate a combined resistance to many antibiotics, thus limiting treatment options [268,269] (Figure 9).

*Pseudomonas* sp. can be frequently present in nosocomial infection and can have severe consequences for immune-compromised people. These infections can be localized in: circulatory system, lung, soft tissue, after burns, complicated UTI and abdomen, cardiovascular system or brain, or they can be related to the medication application system (catheter and surgical wounds). MDR *Pseudomonas aeruginosa* falls into the category of germs which pose a “serious” threat [272].

Recommendations for treatment of MDR *Pseudomonas aeruginosa* include combinations such as: ceftazidime-colistin, ceftazidime-avibactam, ceftolozane-tazobactam, ceftolozane-tazobactam, meropenem, levofloxacin, fosfomycin-colistin, macrolides-tobramycin-trimethoprim-rifampin, imipenem-tigecycline-amikacin, polymyxin-aminoglycoside, cefepime-tazobactam, imipenem-amikacin-cefepime, tigecycline-amikacin-cefepime [272,273,274,275,276,277,278,279,280,281].

## 4. Perspectives in Diminishing Antimicrobial Resistance

The antimicrobial resistance issue can be addressed by combining two or more antibiotics. Their different mechanisms of action provide a higher efficacy. Lately, the research on antimicrobial resistance has been conducted in many directions: bacteriophages, antimicrobial peptides, metal nanoparticles, combinatorial treatment, antibiotic hybrids, etc. [282,283,284].

### 4.1. Bacteriophages

The use of bacteriophages in both prophylaxis and curative treatment against drug-resistant bacteria has emerged as an alternative to antibiotics [285,286].

Bacteriophages are viruses present in all ecosystems, capable of infecting and destroying bacteria, having a significant impact on microbial communities, including on bacterial ecology, decreasing the AMR phenomenon [287]. The mechanism by which bacteriophages infect bacteria is the delivery of the DNA of the phages or even foreign DNA into bacterial cells, thus adding genes to already compromised bacterial genomes [288].

**Figure 10:**
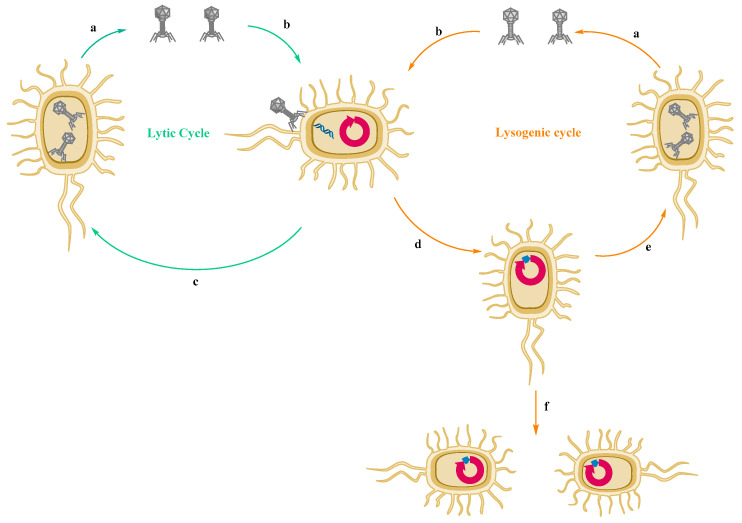
Phage replication cycles: a—bacteria lysis; b—infection; c—replication; d—integration; e—induction; f—vertical transfer. Adapted from [289], published by *Trends Microbiol*, 2018.

DNA or RNA genome of the phages is encapsulated in a protein capsid and can additionally be supplemented by a tail that attaches to targeted bacterial surface receptors and then injects its own genome into bacterial cells, with the appearance of modulations, as follows: modification of bacterial metabolic metabolism for synthesis of the viral proteins and copying of the viral genome. Once the viral particles are assembled, the bacterial cell is lysed. As a result of this process, numerous new phages are released [289]. Figure 10 shows this mechanism schematically.

Bacteriophages have several advantages over conventional antibiotics: (i) the most important is their specificity, because their action usually targets a single bacterial species, leaving the host microbiome unaffected; (ii) bacteriophage replication depends on the presence of the host bacteria and as a result has a self-limiting character [290]. Thus, the main concerns for the therapeutic use of bacteriophages are the possibility of transferring virulence or antibiotic resistance genes, which requires a thorough knowledge of the genomes of these species [291].

In addition to major advantages, there are many others that make it an important strategy in finding new ways to combat AMR and MDR, such as:

- The ability of phages through various self-replication mechanisms to increase their number where their host is present and self-dosing, which prevents the need for repeated administration of phages at the site of action [292];
- They remain in the environment in which they were inoculated as long as the host exists. When all the bacteria have been lysed, the respective phages will also disappear [293];
- Closely related to their specificity is the mechanism of action which is different from antibiotics and thus addresses MDR bacterial species [294,295];
- Phages can be used alone or together with other therapeutic agents (antibiotics, vaccines or various proteins) [285,289];
- Unlike antibiotics, phages are effective both in preventing the biofilm formation and in eliminating bacterial biofilms [296];
- Phages have an important adaptive characteristic so that they can evolve and be able to have an action on bacteria in infecting and lysing them and can adapt to resistant strains [297,298].

Several disadvantages that limit the use of bacteriophages in therapy have been reported, among which: the lack of strain-specific antibacterial activity, low efficacy due to destruction triggered by the immune system and pharmaceutical formulation development difficulties [299,300,301]. The main disadvantage of bacteriophages is the emergence of resistant mutant bacterial species, resulting mainly from the denaturation of bacterial protein structures (lipopolysaccharides, outer membrane proteins), which are not always essential for bacterial survival. One approach to removing this shortcoming is to make combinations between bacteriophages and some antibiotics [302,303].

To avoid therapeutic failure in this new approach, the concept of personalized therapy by using appropriate bacteriophages for their activity against bacteria isolated from infected patients was highlighted. This approach allows the precise targeting of the invading pathogen, while representing the basic concept in “precision medicine” [304,305].

The fundamental characteristic of bacteriophages, to eliminate pathogenic bacteria targets without adversely affecting the microbiome, is the approach of personalized medicine. In the present, researchers focus on the use of bacteriophages for the treatment of MDR infections. This conclusion is based on positive scientific reports of experimental cases, as well as several clinical studies launched worldwide [306,307,308].

### 4.2. Antimicrobial Peptides

Antimicrobial peptides (AMPs) are small peptides (10 up to 60 amino acid residues) widespread in nature playing a key role in the immune system of mammals, amphibians, microorganisms, insects, etc. The majority of AMPs are cationic and their action mechanisms include membrane targeting and non-membrane targeting (membrane permeabilization, inhibition of intracellular functions, immunomodulatory activity, disassembly of biofilms, etc.). Many AMPs proved to be active on ESKAPE pathogens at low MICs. The presence of metal ions, the pH and enzymes (especially proteases) may influence antimicrobial activity of AMPs. Currently, many AMPs are subject to clinical studies and some of them are already approved by FDA (gramicidin, daptomycin, colistin, vancomycin, dalbavancin, telavancin, etc.). The use of AMPs is limited due to their susceptibility to hydrolytic degradation, lack of specifics, poor bioavailability, short half-lives, toxicity and high production costs. More studies are needed in order to obtain increased activity of AMPs and to modulate their absorption, distribution, metabolism, excretion and toxicity (ADMET) properties [309,310,311,312].

### 4.3. Metal Nanoparticles and Metal-Nanoparticle-Based Combinatorial Treatments

Another therapy alternative to treat mainly ESKAPE bacteria is represented by nanoparticles with metals (e.g., silver, gold, etc.) or metal oxides (e.g., zinc oxide, titan dioxide, etc.). These compounds increase cell permeability through disrupting of the cell membrane, release metal ions and interact with DNA or sulfur- and phosphorous-containing compounds and have some advantages such as: limited risks compared to other antibiotics (e.g., adverse reactions, AMR, etc.), control delivery, large therapeutic window, etc. On the other hand, these compounds are not long-term studied and have moderate stability in biological fluids and presented an under-optimal metal ions release. The stabilizing of metal nanoparticles could be realized with proteins, nucleic acids and polysaccharides used as biopolymers. Furthermore, metal-nanoparticle-based combinatorial treatments with antibiotics improve the antimicrobial activity, have a better efficiency (including against MDR bacteria) due to the synergism of action at lower antibiotic doses, and present a lower risk of toxicity or antibiotic resistance [313,314].

### 4.4. Antibiotic Hybrids

Antibiotic hybrids are covalent structures between two antibiotics with different mechanism of action or between an antibiotic and an adjuvant such as efflux pump inhibitor (e.g., naringenin, quercetin, kaempferol, chrysin and genistein, etc.) or siderophore (iron carrier) used to facilitate the access to the target or to increase the antibiotic efficacy. The combination of iron-chelating siderophore with biocidal pharmacophore is named “Trojan horse strategy” and allows the hijacking of the bacterial iron transport system and increases the drug concentration inside the cell (e.g., cefiderocol derived by ceftazidime and catechol 2-chloro-3,4-dihydroxybenzoic acid) [284,315].

The covalent link could be cleavable into two independent molecules in pro-drug structures (e.g., cefamandole derivative linked to omadine) or could be non-cleavable in antibiotic hybrid drugs (e.g., cadazolid-containing ciprofloxacin and tedizolid). In this case, the structure represents a single compound with a specific mechanism of action [316].

Currently, the most hybrids studied contain: (i) fluoroquinolones (hybrids of ciprofloxacin with trimethoprim, naringenin a flavonoid, neomycin, pyrazinamide, tobramycin; 4H-4-oxoquinolizine with rifampicin pharmacophore; etc.); (ii) tobramycin (e.g., hybrids with lysine peptoid mimic, paroxetine); (iii) fluoroquinolone and tobramycin (e.g., moxifloxacin-tobramycin hybrid, ciprofloxacin-tobramycin hybrid etc.) [284,316].

Even if the research in this field is promising, some major difficulties have been identified: (i) the pharmacokinetics could be non-complementary, (ii) the combination ratio different form 1:1 is unavailable, (iii) the designing of the bacterium-specific cleavable linker that is stable and capable of withstanding human metabolic enzymes is needed; (iv) the adequate permeability Gram-negative bacteria, etc. [284].

### 4.5. Guidelines for Rational Use of Antibiotics

Because of AMR high incidence and risk, all stakeholders should contribute to the fight against this major problem. Many institutions or professional associations have published guidelines against AMR. For example, the Infectious Diseases Society of America elaborated some guidelines regarding the treatment of antimicrobial resistance. In this document, guidelines on the treatment of hospital infections caused by some Gram-negative bacteria resistant to antibiotics with significant morbidity and mortality are provided [317].

Another institutional organism involved in this fight is The World Organization for Animal Health. Its main objectives are improving quality of veterinary education worldwide (including in the fields of microbiology, pharmacology and ethics), international cooperation, ensuring the animal health surveillance and a rapid response to contain outbreaks at source, etc. [318].

Despite the large number of these recommendations, many of them were not considered. Thus, the decision-makers should be more involved in the analyzing and reporting of AMR cases [319].

Lately, the authorities and professional associations introduced guidelines in order to determine an adequate use of antibiotics in hospitals. These contain several strategies, such as [320,321,322]:

- Optimization and even decrease of antibiotic prescriptions,
- Usage of targeted antibiotics based only on the clinical and microbiological diagnostic (the transition from empirical therapy to targeted therapy),
- Reporting, collecting and analyzing of data regarding antimicrobial susceptibility and antibiotic consumption,
- Optimization of doses, treatment duration and dosing time intervals, according to the nature and severity of the infection, including the usage of biomarkers (e.g., procalcitonin),
- Parenteral-to-oral conversion if sufficient bioavailability is assured,
- Reduction of routine use for some antibiotics (e.g., cephalosporins, fluoroquinolones, etc.) in favor of others (e.g., penicillin), etc.

## 5. Conclusions

The emergence of AMR is considered one of the most important challenges of the 21st century. An increased risk of morbidity and mortality is associated with infections caused mainly by ESKAPE pathogens that have become resistant to one or more antibiotics. The study of these emerging microorganisms and the mechanisms by which they develop resistance as well as comprehensive knowledge of the effective therapeutic options could help to minimize the pace of AMR. Besides various political-legislative measures, in order to reduce the spread of AMR, it is recommended to avoid automedication and the unnecessary prescription of antibiotics, as well as giving up their misuse. Furthermore, novel antibiotics or alternative therapies are needed in order to address the problem of AMR.

## Figures and Tables

**Table 1:** Classification of antibiotics according to their mechanisms of action.

Mechanism of Action	Antibiotic Class	Reference
Inhibition of bacterial cell wall synthesis	Penicillins Cephalosporins Monobactams Carbapenems Glicopeptides Polypeptides	[21,25,26]
Depolarization of the bacterial cell membrane	Lipopeptides antibiotics	[27]
Inhibition of protein synthesis: Binding to 30S ribosomal subunits	Aminoglycosides Tetracyclines	[28,29]
Inhibition of protein synthesis: Binding to 50S ribosomal subunits	Macrolides Amphenicols Lincosamides Streptogramins Oxazolidindiones	[30,31,32]
Inhibition of DNA synthesis	Quinolones Fluoroquinolones Nitroimidazoles	[33]
Inhibition of RNA synthesis	Rifamycins	[34]

**Table 3:** The prioritization of microorganisms in terms of their pathogenicity. Adapted from [122], published by *Can J Microbiol*, 2019.

Pathogen	Resistance Type	Competence
CRITICAL PRIORITY		
*Acinetobacter baumannii*	carbR	Natural competence
*Pseudomonas aeruginosa*	carbR	Natural competence
*Enterobacteriaceae*	carbR, cephR	Predicted natural competence
(*Klebsiella pneumonia*, *Escherichia coli*, *Enterobacter* spp., *Serratia* spp., *Proteus* spp., *Providencia* spp., *Morganella* spp.)		
HIGH PRIORITY		
*Enterobacteriaceae*: *Salmonella* spp.	flrqR	Predicted natural competence
*Staphylococcus aureus*	vanR	Natural competence
*Helicobacter pylori*	clarR	Natural competence
*Enterococcus faecium*	vanR	-
*Neisseria gonorrhoeae*	flrqR, cephR	Natural competence
*Campylobacter* spp.	flrqR	Natural competence
MEDIUM PRIORITY		
*Enterobacteriaceae*: *Shigella* spp.	flrqR	Predicted natural competence
*Streptococcus pneumoniae*	penR	Natural competence
*Haemophilus influenza*	ampR	Natural competence

Abbreviations: carbR = carbapenem-resistant; flrqR = fluoroquinolone-resistant; vanR = vancomycin-resistant; clarR = clarithromycin-resistant; cephR = 3rd-generation cephalosporins-resistant; ampR = ampicillin-resistant; penR = penicillin-resistant.

## Data Availability

Not applicable.

## Abbreviations

ADMET	absorption, distribution, metabolism, excretion and toxicity
AMK	amikacin
AMPs	antimicrobial peptides
AMR	antimicrobial resistance (AMR)
ARCD	antibiotic-resistant *Clostridium difficile*
attI	recombination site
AZT	aztreonam
BSI	bloodstream infection
CAZ	ceftazidime
CDI	*Clostridium difficile* infections
CFM	cefixime
CFZ	cefazolin
CPM	cefepime
CRE	carbapenem-resistant *Enterobacteriaceae* spp.
CREC	carbapenem-resistant *Escherichia coli*
CRKP	carbapenem-resistant *Klebsiella pneumoniae*
CTX	cefotaxime
EPS	extracellular polymeric substances
ESBL	extended spectrum beta-lactamases
ETP	ertapenem
FQ	fluoroquinolone
GEN	gentamicin
HGT	horizontal gene transfer
IMI	imipenem
intI	integrase gene
IS	insertion sequences
LGT	lateral gene transfer
MDR	multidrug-resistant
MEM	meropenem
MGEs	mobile genetic elements
MIC	minimum inhibitory concentration
MRG	macrolide resistance genes
MRSA	methicillin-resistant *Staphylococcus aureus*
PBP	Penicillin-binding proteins
PDR	pan-drug-resistant
PTZ	piperacillin-tazobactam
rCDI	relapse after *Clostridium difficile* infection
SSI	surgical site infection
TEs	transposable elements
TET	tetracycline
TMT-SMX	trimethoprim-sulfamethoxazole
Tn	transposons
TOB	tobramycin
UTIs	urinary tract infections
VISA	*Staphylococcus aureus* intermediate-resistant to vancomycin
VRE	vancomycin-resistant enterococci
VRSA	vancomycin-resistant *Staphylococcus aureus*
XDR	Extensively drug-resistant
